# The feasibility and acceptability of neuromuscular electrical stimulation to improve exercise performance in patients with advanced cancer: a pilot study

**DOI:** 10.1186/1472-684X-13-23

**Published:** 2014-05-01

**Authors:** Tamara Windholz, Tara Swanson, Brandy L Vanderbyl, R Thomas Jagoe

**Affiliations:** 1 Segal Cancer Centre, Jewish General Hospital, 3755 Cote Ste Catherine, H3T 1E2 Montreal, Quebec, Canada

**Keywords:** Neuromuscular electrical stimulation, Exercise training, Performance status, Cancer rehabilitation

## Abstract

**Background:**

To determine the feasibility and acceptability of lower limb neuromuscular electrical stimulation (NMES) as a home-based exercise therapy in patients with cancer who could not attend hospital-based exercise training.

**Methods:**

A single-arm prospective pilot study of NMES, applied daily to both quadriceps muscles for six weeks. Participants were recruited from patients referred to a hospital-based multi-disciplinary supportive care team specializing in treatment of patients with nutritional depletion and functional decline.

**Results:**

Of the 15 participants who underwent baseline testing, 10 (67%) completed the study and only one (7%) withdrew because of discomfort due to NMES treatment. 7/10 (70%) of participants used NMES at least three times a week for the duration of the study. Use of NMES did not lead to significant improvements in physical performance tests.

**Conclusions:**

NMES is a feasible and acceptable intervention for home use in patients with cancer, poor performance status and metastatic disease. However, whether NMES is an effective strategy to stabilize or improve physical performance in such patients is not proven.

## Background

Patients with cancer frequently suffer problems common to other chronic diseases, namely long-lasting sequelae from their disease and its treatments [1] as well as progressive decline in overall functional status [2,3]. In many chronic medical conditions e.g. chronic obstructive pulmonary disease (COPD), and congestive heart failure (CHF), exercise-based rehabilitation programs have a major impact on quality of life and functional status [4] and are widely deployed as part of standard clinical care. However, despite growing evidence that exercise training in cancer patients also contributes to improved quality of life and tolerance of anti-cancer treatment [5], exercise-rehabilitation has not been generally adopted for patients with cancer.

Exercise training programs for patients with chronic diseases typically involve hospital-based supervised training two or three times a week. However, attendance at hospital-based exercise training is difficult for debilitated patients with cancer; and though patients with advanced cancer are willing to exercise they strongly prefer to do so at home [6,7]. However the beneficial impact of such home-based training is still unclear and is difficult to measure. For example, a recent study of home-based semi-supervised exercise was unsuccessful because of unexpected poor accrual and high dropout rates [8]. Outside of such studies, community or home-based supervised training is often not readily available for patients with medical conditions. Thus by default, a self-directed unsupervised home exercise program is often recommended for patients unable or unwilling to attend hospital training, despite the inherent difficulties in monitoring exercise adherence and intensity. Other approaches are therefore needed which can deliver a safe and effective exercise stimulus at home, in a manner which is suitable for use by patients with poor physical function.

Neuromuscular electrical stimulation (NMES) delivers a controlled contractile stimulus to underlying muscles via surface electrodes placed on the skin. It is widely used for rehabilitation of neurological or orthopedic conditions. NMES is often used to assist with rehabilitation following joint surgery [9], by helping to speed up the recovery of muscle strength and joint function. Moreover, NMES used in conjunction with voluntary contraction exercises, accelerates leg muscle strengthening after knee arthroplasty [10]. More recently NMES has been assessed for patients suffering chronic medical conditions. In debilitated patients with COPD or CHF, NMES of the large lower limb muscles was shown to increase muscle size [11], improve muscular strength and overall exercise capacity [12-14], facilitate activities of daily living, and improve quality of life [15].

Despite the evidence for effectiveness of NMES in other patient populations, relatively few studies have been performed to test the potential usefulness of NMES in patients with cancer. One case study reported use of NMES in a single patient with extensive metastatic lung cancer, who achieved improvements in mobility, function and quality of life with NMES [16]. Another pilot study used NMES in a randomized trial involving 16 advanced stage non-small cell lung cancer patients with good performance status. Those receiving NMES found it highly acceptable, but there was only a non-significant trend towards improvement in leg muscle strength or other measures of endurance and habitual daily activity [17]. In a follow-up phase II study, the same authors recruited lung cancer patients undergoing first-line palliative chemotherapy and randomized them to NMES intervention (30 patients) or a control (19 patients) group [18]. In this later study there was no improvement in the physical function tests (quadriceps strength or physical activity), and a lower than expected level of adherence. As a result, the authors concluded that NMES does appear to warrant further study in advanced lung cancer patients at this stage in their treatment [18]. This does not rule out a role for NMES to reduce muscle loss and dysfunction [19] in other patients with cancer, but to date there is still little published data on the use of NMES in patients with cancer in general, especially those with poor performance status.

The McGill Cancer Nutrition-Rehabilitation Program clinic at the Jewish General Hospital (CNR-JGH clinic) in Montréal, is a multi-disciplinary team that specifically addresses the clinical needs of patients with cancer referred with weight loss, anorexia, and reduced physical function. The CNR-JGH clinic uses a comprehensive interdisciplinary approach to assess and control symptoms, along with individualized dietary interventions and exercise training wherever possible. In our experience, hospital-based exercise training is very difficult to deliver to patients with poor performance status and those who live far away from the hospital. To address this practical problem, a pilot study was performed to evaluate the acceptability and feasibility of a home-based NMES intervention in a heterogeneous group of patients attending the CNR-JGH clinic, the majority of whom had advanced stage cancer. The secondary objective of the study was to assess the impact of the NMES intervention on tests of physical function focused on walking endurance capacity, lower extremity strength and global functional performance.

## Methods

### Patient recruitment

Patients were recruited from those referred to the CNR-JGH clinic for consultation by their treating oncology teams at the Jewish General Hospital: an urban university teaching hospital in Montréal, Québec, Canada. The participants recruited included both those with impaired performance status (PS = 2 or 3) [20] and patients with better performance who were unable to attend hospital-based supervised physical rehabilitation more than once a week due to travel distance or other similar constraints. Patients were excluded if they had any cognitive impairment affecting their ability to apply NMES safely without medical supervision, skin conditions contraindicating repeated use of surface electrodes, orthopedic implants in the hip joint, metastatic lesions to the femur, or electronic implants (e.g., pacemakers and defibrillators). Patients who were already actively participating in a supervised rehabilitation or exercise intervention greater than once a week were also ineligible.

### Baseline functional assessment

All assessments, instruction and follow-ups were performed by the study physiotherapist in the CNR-JGH clinic. Baseline functional evaluation was performed as follows: a) Walking endurance capacity was assessed with the 6-minute walk test (6 MWT) [21]. If patients were unable to walk independently even with standard walking devices (including rolling walking frames) a score of 0 m was recorded. b) Lower extremity functional strength was assessed using the repeated Sit-to-Stand (STS) test, as included in the Simmonds Functional Assessment Battery [22]. The patient was instructed to go from sitting to standing twice in a row as quickly as possible, without the use of their arms. The test was performed twice and the average time of the two trials was recorded. c) Global functional performance status (PS) [20] was recorded based on patient’s report of their level of physical activity to the study physiotherapist.

### Instruction and use of neuromuscular electrical stimulation (NMES) machine

Participants were instructed on the application of the surface electrodes and the use the portable NMES machine (Neurotech MediStim, Galway, Ireland). Initial training was performed to ensure that participants were able to apply the electrodes and use the device on themselves without additional assistance. After cleaning the skin with an alcohol pad, disposable electrodes (EMPI Self-adhesive Carbon FM electrodes 2”x 2”) were applied to the motor points of the Vastus Medialis Oblique distally, and over the midpoint of the quadriceps muscle belly more proximally [23]. The electrodes placed on each thigh were then connected to the individual leads of the NMES unit, to allow for separate intensity control for each quadriceps. Participants were instructed to use NMES at home for 6 weeks on both quadriceps muscles simultaneously for 30 minutes a day. Stimulation parameters were as follows: 300 ms pulses, 50 Hz, alternating 5 s on 5 s off (50% duty). Stimulation intensity was individually adjusted for each limb in order to obtain tetanic contraction or maximum tolerated intensity. In addition, participants were instructed to voluntarily contract the quadriceps muscles during the periods of NMES stimulation (active co-contraction) to enhance the strengthening effect and improve tolerance of NMES. Participants were positioned with their legs on a horizontal surface, and the knee supported on a rolled towel. They were asked to perform an isometric contraction of the quadriceps for the duration of the stimulus, by tightening the thigh muscle and pushing the knee down on towel without lifting the foot. Where appropriate, participants could progress the co-contraction exercise if their strength improved. For this second stage they were instructed to raise their leg off the surface and completely extend the knee against gravity.

### Study completion and adherence with NMES intervention

The study physiotherapist called or met with each participant at the mid-point (3 weeks) of the study to give additional instruction if needed. Each patient had a maximum of 6 weeks (42 days) of NMES and the total (days) and % maximum possible adherence were calculated using the logs of daily NMES use completed by each patient. Study completion was achieved if patients completed the assessment at the end of the intervention period. Patient adherence was measured using an intervention diary in which participants were asked to log their use of the NMES machine daily, and document any variance from the prescribed intervention. The percentage of maximum possible adherence was calculated using the total number of days during the intervention period when the patient could have used the NMES machine. Thus, if the patient was hospitalized for 7 days during the study, the % maximum possible adherence was calculated as a proportion of 35 days not 42.

### Outcome assessments

#### Primary outcomes

Feasibility was assessed by measuring the proportion of patients who completed the 6-week intervention, and by the overall level of adherence achieved over the intervention period. Acceptability of NMES was evaluated at the end of the 6 weeks by a brief structured questionnaire developed for this study which was completed by each patient. The questionnaire used a 5-point Likert scale to rate their answers from 1 = “strongly disagree”, 2 = “disagree”, 3 = “neutral”, 4 = “agree”, 5 = “strongly agree”.

#### Secondary outcomes

Physical performance measures were performed at baseline (PS, 6 MWT, STS) and at the end of the study. To determine the effects of adherence with NMES on physical function parameters, results were compared in patients with <40% (poor) vs >40% NMES adherence.

### Ethics

The study was approved by the Jewish General Hospital Research Ethics Committee and all participants provided written informed consent.

### Statistical analysis

Comparison between participants who did and did not complete the study was performed using unpaired t-test and Fisher’s exact test for continuous and categorical variables respectively. Assessment of change from before and after NMES intervention was performed using paired t-tests or Fisher’s exact test for PS category count data. To assess factors potentially relevant to adherence and change in physical performance tests, mean differences were compared using t-tests and differences in counts within categories were assessed using Fisher’s exact test. All analyses were performed using R [24].

## Results

**Figure 1:**
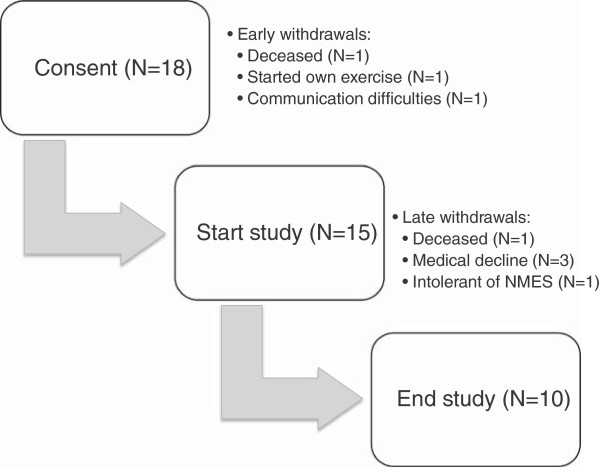
Breakdown of reasons for early and late withdrawals from the NMES study.

111 new patients were seen in the CNR-JGH clinic between June 2010 and July 2011. Of these 52 (47%) were potentially eligible for the study (poor performance status and/or inability to attend hospital-based exercise training). After further screening, 37 patients were approached and of these 18 (49%) patients were recruited. The reasons that 19 patients were not consented for the study ranged from medical deterioration (3) or improvement (1), preference for standard exercise (4), feeling that the study was not of interest or too burdensome (6), and follow-up and communication difficulties with study coordinator (5). Of the 18 patients who gave signed consent, three withdrew from the study and did not proceed to baseline assessment (Figure 1). Reasons for early withdrawals included: rapid medical deterioration and death (n = 1), started exercising regularly at a local gym (n = 1), withdrawn due to language and communication difficulties which became evident only after consent was obtained (n = 1). The remaining 15 patients underwent baseline function testing and their demographic and disease characteristics are shown in Table 1. Two patients with good PS (PS = 1) were also included in the study as they were not exercising regularly and could not attend the usual hospital-based supervised training sessions. The majority of patients (73%) had metastatic (stage IV) disease. The most common tumour types were lung or gastrointestinal cancers, but five patients had other types of tumours including breast (n = 1), ovarian (n = 1), endometrial (n = 1) and haematological (n = 1) malignancy. One additional patient was being treated with cyclical intravenous chemotherapy for systemic amyloidosis without overt evidence of underlying malignancy. Overall 67% of the patients were undergoing active treatment with chemotherapy and 40% were taking, or had completed within the preceding 14 days, an extended (≥3 weeks’ duration) course of corticosteroids (Table 1). Results of functional testing at baseline are shown in Table 2. As a group, the patients in this study had impaired exercise capacity with mean 6 MWT of 257 m (50.2% predicted) and mean STS time of 8.0 s, which is 2.6 standard deviations above the mean expected in (i.e. slower than) healthy individuals of similar age (Table 2).

**Table 1:** Demographic and disease characteristics of patients who underwent full baseline assessment

		**All subjects (N = 15)**		
		**Completed (N = 10)**	**Withdrawn (N = 5)**	
	**Mean (SD)**			**P**
**Age yrs**	67.9 (9.4)	67.6 (10.9)	68.4 (6.3)	0.88
**Body mass index kg/m**^**2**^	23.1 (4.6)	23.7(4.9)	22.1(4.2)	0.52
	**N (%)**			
**Sex**				
M	9 (60)	4 (40)	5 (100)	
F	6 (40)	6 (60)	0 (0)	0.04
**PS**				
1	2 (13)	2 (20)	0 (0)	
2	6 (40)	4 (40)	2 (40)	
3	7 (47)	4 (40)	3 (60)	0.79
**Diagnosis**				
Lung cancer	4 (27)	3 (30)	1 (20)	
GI cancer	6 (40)	2 (20)	4 (80)	
Other	5 (33)	5 (50)	0 (0)	NA
**Cancer stage**				
III	1 (7)	1 (10)	0 (0)	
IV	11 (73)	6 (60)	5 (100)	
NA	3 (20)	3 (30)	0 (0)	NA
**Chemotherapy**				
Y	10 (67)	6 (60)	4 (80)	
N	5 (33)	4 (40)	1 (20)	0.60
**Recent steroid use**				
Y	6 (40)	4 (40)	2 (40)	
N	9 (60)	6 (60)	3 (60)	1.00

Notes: P indicates result of significance testing (unpaired t-test for means or Fisher’s exact test for count data) comparing patients who did, or did not, complete the study.

**Table 2:** Physical performance evaluation results at baseline and after six weeks of NMES intervention

				**Baseline**			**End of study**		
				**All subjects (N = 15)**					
				**Withdrawn (N = 5)**	**Completed (N = 10)**	**P**^**a**^	**Completed (N = 10)**	**Difference**	**P**^**b**^
	**Number**								
**PS**	1			0	2	0.79^#^	4		0.15^#^
	2			2	4		4		
	3			3	4		2		
	**Mean (SD)**								
**6 MWT**	m		257 (160)	166 (138)	303 (157)	0.11	282 (171)	−21.1 (167.7)	0.70
	%		50.2 (32.2)	31.0 (24.6)	59.8 (32.2)	0.08	56.0 (34.5)	−3.8 (33.3)	0.73
**STS**	s		8.0 (4.3)	7.6 (3.4)	8.2 (4.8)	0.80	7.0 (3.3)	−1.2 (3.4)	0.30
	S-score		2.6 (2.4)	2.2 (2.0)	2.7 (2.2)	0.69	2.0 (1.8)	−0.7 (2.0)	0.30

Notes: Performance status (PS), six-minute walk distance (6 MWT) in metres (m) and expressed as % predicted (from [21]), Sit-to-stand (STS) test expressed as seconds (s) and as a standard score (S-score) value calculated using age range-specific mean and standard deviations for healthy controls (from [22]): positive scores indicates STS S-scores above the mean. P^a: #^Fisher’s exact (for counts) or unpaired t-test (for means) comparing baseline results for patients withdrawn and patients who completed study. P^b : #^Fisher’s exact (for counts) or paired t-test (for means) comparing baseline and final test results for patients who completed the study.

### Feasibility

**Figure 2:**
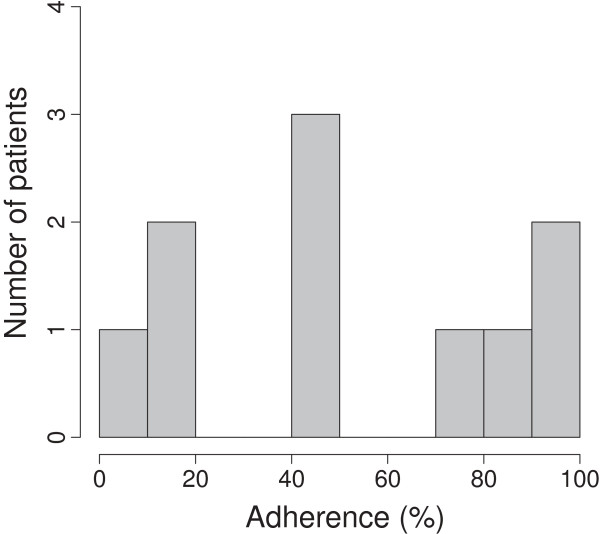
Histogram of adherence with NMES intervention during study.

Completion of the study was achieved by 10 (66.6%) of the 15 patients. All 15 had baseline assessments, used the NMES for the 6-week intervention period, and completed the final assessments. Four of the five late withdrawals were not due to the NMES intervention, namely: death (n = 1) and deterioration in medical status (n = 3). Only one patient withdrew because they found they could not tolerate the local discomfort due to repeated NMES treatments (Figure 1). For those completing the study the machine was used for mean(SD) 20.1(13.0) days during the 42-day study period. When the adherence was adjusted to account for periods when NMES had to be stopped during hospital admissions, this showed that patients used their machines over half the available time (mean(SD) 54.2(32.8)%). On closer examination there were in fact three groups: three poorly adherent patients (mean(SD) use 14.3(6.3)%), three moderately adherent patients (mean(SD) use 49.4(1.1)%) and four highly adherent patients (mean(SD) use 87.8(8.7)%) (Figure 2). Thus, 7(70%) patients completing the study achieved more than 40% adherence: equivalent to NMES use at least three times a week. In subsequent analysis to explore the potential impact of adherence on physical function two groups were defined: poorly adherent (<40%, n = 3) and adherent (>40%, n = 7) patients.

### Acceptability

Informal feedback during the study was positive about the use of NMES at home. Furthermore, the acceptability of NMES at a group level was captured using the questionnaires at the end of the study. More than 50% of patients felt strongly that 6 weeks’ of NMES use was acceptable (Table 3: C). Furthermore, a majority of patients reported that NMES was a useful in helping with activities and symptoms (Table 3: F, G). In addition 50% of patients were interested in using NMES outside of a study (Table 3: H).

**Table 3:** Responses to feedback questions for patients completing the study

	**Question text**	**Median**	**Range**
**A**	“NMES was helpful for me”	4	2-5
**B**	“NMES is something patients can be taught to use safely at home”	5	3-5
**C**	“Using the NMES for 6 weeks is acceptable”	5	2-5
**D**	“Using the NMES for 30 mins a day is acceptable”	4	2-5
**E**	“Home NMES was a good intervention for me”	4	2-5
**F**	“Using NMES had a positive impact on my daily activities”	4	2-5
**G**	“Using NMES had a positive impact on my symptoms”	4	2-5
**H**	“If offered outside of a study, I would want to use NMES”	3	2-5

Notes: Allowed responses ranged from 1–5 where 5 = “totally agree” (see Methods).

### Changes in physical function

Baseline and final assessment results were compared for Completers (Table 2). Global performance status and STS appeared somewhat better, but these changes did not achieve statistical significance (Fisher’s exact test p = 0.15 comparing categories of PS; mean reduction in STS = −1.2s, P = 0.3). Only one patient was unable to perform the 6 MWT at baseline, but they had improved and walked 220m at end of study. However, as a group there was no change in mean 6 MWT distance after the NMES intervention period (mean difference = −21.1m, P = 0.7) (Table 2). Adherence with NMES might be expected to be a major determinant of improvement in physical function, but we were not able to detect any such effect of adherence with the number of patients recruited in this study. Thus, the patients with poor adherence (<40%, n = 3) did not have significantly different changes in STS or 6 MWT compared to the adherent patients (>40%, n = 7) (P = 0.56, 0.71 respectively)*.* It was also noted that those on long term steroids had significantly reduced 6 MWT at beginning of study (mean(SD): recent steroids 166(139)m vs no steroids 394(88)m, P = 0.04). We hypothesized that NMES may have a particularly useful role in promoting improving function in these patients. However despite a trend towards differential improvement in patients on steroids, this did not reach statistical significance (6 MWT mean(SD) change: recent steroids 35(184)m, no steroids −59(161)m, P = 0.4). A test of proportions gave similar suggestive but non-significant results for both change in 6 MWT and PS, e.g. for PS: 3 out of 4 patients who had recently been on an extended course of oral corticosteroids reported improved PS compared with only 1 out of 6 non-steroid-treated patients (Fisher’s exact test, P = 0.19).

## Discussion

This is the first report of the use of NMES in patients with advanced cancer and poor performance status or other barriers to usual exercise training. Our results suggest that NMES is feasible and well-tolerated in this group of patients with advanced cancer who are unable to attend hospital-based exercise training. Only one patient (7%) withdrew from the study because they were unable to tolerate the NMES intervention, whereas four patients (27%) withdrew or could not complete the study, because of medical complications or death. Furthermore, qualitative feedback confirmed that home-based NMES is highly acceptable. There was a wide range in adherence with NMES over the 6-week period of intervention, but despite this, 70% of patients used the NMES for at least 40% of the available days. This equates to use at least three times a week, which is the usual frequency for standard exercise training programs. Repeated physical performance testing before and after NMES revealed wide intra-individual variability. However, there was no improvement in objective measurements of exercise function after NMES intervention (Table 2). Furthermore, no dose-effect of NMES was demonstrated, as there were no differences in physical functional outcomes, between adherent (>40% maximum usage) and poorly adherent (<40% maximum NMES usage) patients.

We recognize a number of limitations to interpreting our results, including the small number of analyzable patients (10) in this pilot study. The primary study objective was to assess feasibility and acceptability of NMES in a variety of cancer patients and for this reason the patients recruited for the study had a range of pathological types and stages of cancer. However, as a result the study participants also had a widely varying initial functional status and exercise performance, making it more difficult to reliably detect functional changes attributable to NMES. We did not include an untreated control group to assess the likely spontaneous changes in outcome measures over the same time period. In debilitated advanced cancer patients, progressive physical deterioration may be anticipated. Thus a reasonable minimal expectation of successful NMES, and other exercise interventions, would be to inhibit this deterioration, rather than achieve an improvement from baseline. Without an untreated comparison group we are unable to say whether there was any indication of this beneficial effect of NMES in the current study. Due to limited personnel resources, the same physiotherapist who instructed the patient in the use of NMES also performed the outcomes assessments. This raises the possibility of patient response bias, particularly for reported global performance status and responses to questionnaire items.

Maddocks et al. [18] reported only 15/30 lung cancer patients receiving palliative chemotherapy completed approximately 10 week NMES intervention. In contrast to the present study, they concluded that NMES was not an acceptable treatment based on a pre-defined 80% group adherence threshold [18]. However it is notable that in line with our results only 3(11%) of their patients withdrew because of NMES discomfort suggesting that the intervention, per se, is tolerable for the majority. Furthermore, without any correction for periods when patients were hospitalized etc., 50% of patients in the study by Maddocks et al. achieved the required three times a week treatment frequency, and another 3 patients fell below this threshold because of only one missed session. We would concur with the authors of that study who acknowledged that their conclusion, that NMES is “unacceptable”, may have been based on unrealistically stringent criteria [18].

Published studies of use of NMES describe a range of different electrical stimulation protocols [25] and there is no clear consensus as to the optimal stimulation frequency, electrode placement sites and duty-cycle etc. The original intention in this study was to use dual stimulation of quadriceps and gluteus muscles. However, it became clear that there were both practical and patient acceptability difficulties in repeatedly and consistently placing the gluteus muscle electrodes, especially in very sedentary patients or those requiring help from caregivers to do so. As a result a simplified protocol was used with electrical stimulation of quadriceps only, combined with active voluntary co-contraction of the stimulated limb muscles, to enhance the contractile stimulus. The use of active co-contraction is novel and proved both practical and acceptable as demonstrated by the feedback received (Table 3). Other informal comments received also suggested the patients appreciated the ability to participate in their exercise training even in this limited way.

Our study was not powered to detect differences in the physical outcome measures and many other confounding factors, including acute changes in medical status, can influence the results of the physical performance tests. The physical outcomes measures adopted (PS, STS, 6 MWT) have been widely used in studies of cancer patients and are relevant to normal daily functional activities [22]. However, direct measurements of muscle strength or cross-sectional area, have been used by other authors [11,14,17], and these approaches may be more sensitive to detect modest short-term improvements in muscle induced by NMES. Analysis of pooled data from several different studies of NMES in chronic medical conditions suggests NMES use leads to a mean improvement in 6 MWT of 40 m [26]. However we calculated that 127 patients would be needed to detect a difference of 40 m in 6 MWT (effect size 0.25 SD) with 80% power in any future study, without modifying the entry criteria. This is directly related to the wide variance in initial 6 MWT results. Thus stratifying analysis or selecting patients with a pre-defined baseline performance (e.g. by 6 MWT distance) would make it easier to detect subsequent changes after NMES intervention.

High drop-rate rates and the continuing uncertainty about which patients are most likely to respond to NMES physiologically, present significant challenges to successful completion of studies of NMES in cancer patients. It is noteworthy that many studies in COPD and CHF conclude that use of NMES may be best targeted to the most debilitated patients with very poor function [14]. However cancer patients who have both advanced disease and impaired functional status (e.g. 6 MWT <350 m at baseline [27]) have very poor prognosis, and even short-term studies in these patients are likely to suffer substantial dropouts [3,8] due to medical deterioration and death. In this pilot study we were not able to confidently identify any subgroups of patients who have greater potential to complete the NMES intervention. Thus patients who did not complete the intervention had a trend towards lower mean baseline 6 MWT distance (166 m Withdrawn vs 303 m Completed, P = 0.11) (Table 2). However, neither this nor any other demographic or physical function results at baseline were significantly different between those that completed and those who were withdrawn from the study (Tables 1, 2).

One group of patients who may be more likely to benefit from NMES are those that have taken a recent prolonged course of corticosteroids, given that corticosteroids are both widely used in cancer therapy and can cause myopathy [28]. Consistent with this notion, the data from this study did confirm that those patients recently treated with steroids had poorer function at baseline (6 MWT mean(SD): recent steroids 166(139)m vs no steroids 394(88)m, P = 0.04). However there was only a non-significant trend towards a differential improvement in 6 MWT in patients treated with extended courses of steroids. Nevertheless, further studies of NMES may be warranted to establish if early use of NMES can be of particular benefit in cancer patients treated with prolonged courses of steroids. Similarly, it is conceivable that other subgroups of cancer patients should be considered for more focused studies, including the more elderly patients with comorbidities [29] and those being treated with antitumour agents, such as Sorafenib, which promote muscle loss [30].

## Conclusions

The results of the current pilot study suggest that NMES is both feasible and acceptable in a mixed group of patients with cancer, most of whom had poor performance status. This study does not demonstrate that NMES leads to improved physical functioning in cancer patients with poor performance status despite the success of NMES in other chronic diseases and clinical settings. One reason for the difficulty in detecting functional benefits of NMES in cancer patients may be their marked heterogeneity in terms of medical status and physical functioning. As a result, though it is likely that NMES has a role in achieving or maintaining optimal functional ability in selected patients with cancer, this still remains to be confirmed by future prospective studies. Such studies should be designed to both identify the best candidates for NMES interventions and to confirm the optimal method of delivery of this type of therapeutic exercise.

## Abbreviations

6MWT: Six-minute walk test; CHF: Congestive heart failure; CNR-JGH: The McGill Cancer Nutrition-Rehabilitation Program clinic at the Jewish General Hospital; COPD: Chronic obstructive pulmonary disease; NMES: Neuromuscular electrical stimulation; PS: Performance status; STS: Sit-to-stand test.

## Competing interests

RTJ has received payments as an advisory board member for NEOMED and lecturer fees from Abbott Laboratories. The authors confirm that individuals or groups who have provided funding for the CNR-JGH activities have not been involved in any aspect of the study design, execution or analysis.

## Authors’ contributions

RTJ and TS conceived and designed the study, wrote the protocol and obtained initial ethics approval. TW finalized the study protocol, performed the patient testing and supervised the NMES interventions. BV recruited patients and coordinated the study including completion of study database. RTJ analysed the study results. All authors contributed to the writing of the manuscript. All authors read and approved the final manuscript.

## Authors’ information

TS and TW are physiotherapists who worked in the CNR-JGH team during the study. BV worked as study coordinator with the CNR-JGH team during the study. RTJ is an MD and Director of the CNR-JGH team.

## Pre-publication history

The pre-publication history for this paper can be accessed here:

http://www.biomedcentral.com/1472-684X/13/23/prepub
