# Correction: The feasibility and acceptability of neuromuscular electrical stimulation to improve exercise performance in patients with advanced cancer: a pilot study

**DOI:** 10.1186/1472-684X-13-33

**Published:** 2014-07-04

**Authors:** Tamara Windholz, Tara Swanson, Brandy L Vanderbyl, R Thomas Jagoe

**Affiliations:** 1 Segal Cancer Centre, Jewish General Hospital, 3755 Cote Ste Catherine, Montreal, QC H3T 1E2, Canada

## Correction

In the course of type-setting this article [1] for final publication the presentation of the data in Tables 1 and 2 was altered. To avoid any confusion and to improve the clarity of our results the corrected Tables are included here.

**Table 1:** Demographic and disease characteristics of patients who underwent full baseline assessment

			**All subjects**			
			**(N = 15)**			
				**Completed**	**Withdrawn**	
				**(N = 10)**	**(N = 5)**	**P**
		**Mean (SD)**				
**Age**	(yrs)		67.9 (9.4)	67.6 (10.9)	68.4 (6.3)	0.88
**Body mass index**	(kg/m^2^)		23.1 (4.6)	23.7(4.9)	22.1(4.2)	0.52
		**N (%)**				
**Sex**						
	M		9 (60)	4 (40)	5 (100)	
	F		6 (40)	6 (60)	0 (0)	0.04
**PS**						
	1		2 (13)	2 (20)	0 (0)	
	2		6 (40)	4 (40)	2 (40)	
	3		7 (47)	4 (40)	3 (60)	0.79
**Diagnosis**						
	Lung Cancer		4 (27)	3 (30)	1 (20)	
	GI cancer		6 (40)	2 (20)	4 (80)	
	Other		5 (33)	5 (50)	0 (0)	NA
**Cancer stage**						
	III		1 (7)	1 (10)	0 (0)	
	IV		11 (73)	6 (60)	5 (100)	
	NA		3 (20)	3 (30)	0 (0)	NA
**Chemotherapy**						
	Y		10 (67)	6 (60)	4 (80)	
	N		5 (33)	4 (40)	1 (20)	0.60
**Recent steroid use**						
	Y		6 (40)	4 (40)	2 (40)	
	N		9 (60)	6 (60)	3 (60)	1.00

Notes: P indicates result of significance testing (unpaired *t*-test for means or Fisher’s exact test for count data) comparing patients who did, or did not complete the study.

**Table 2:** Physical performance evaluation results at baseline and after 6 weeks of NMES intervention

			**Baseline**				**End of study**		
			**All subjects**						
			**(N = 15)**						
				**Withdrawn**	**Completed**		**Completed**		
				**(N = 5)**	**(N = 10)**	**P**^**a**^	**(N = 10)**	**Difference**	**P**^**b**^
		**Number**							
**PS**	1			0	2	0.79^#^	4		0.15^#^
	2			2	4		4		
	3			3	4		2		
		**Mean (SD)**							
**6MWT**	m		257(160)	166 (138)	303 (157)	0.11	282 (171)	−21.1 (167.7)	0.70
	%		50.2 (32.2)	31.0(24.6)	59.8(32.2)	0.08	56.0(34.5)	−3.8 (33.3)	0.73
**STS**	s		8.0(4.3)	7.6 (3.4)	8.2 (4.8)	0.80	7.0(3.3)	−1.2 (3.4)	0.30
	S-score		2.6(2.4)	2.2 (2.0)	2.7 (2.2)	0.69	2.0(1.8)	−0.7 (2.0)	0.30

Notes: Performance status (PS), six-minute walk distance (6MWT) in metres (m) and expressed as % predicted (from [21]), Sit-to-stand (STS) test expressed as seconds (s) and as a standard score (S-score) value calculated using age range-specific mean and standard deviations for healthy controls (from [22]): positive scores indicates STS S-scores above the mean. P^a: #^Fisher’s exact (for counts) or unpaired *t*-test (for means) comparing baseline results for patients withdrawn and patients who completed study. P^b: #^Fisher’s exact (for counts) or paired *t*-test (for means) comparing baseline and final test results for patients who completed the study.

## Pre-publication history

The pre-publication history for this paper can be accessed here:

http://www.biomedcentral.com/1472-684X/13/33/prepub
